# Simulation of the effects of COVID-19 testing rates on hospitalizations

**DOI:** 10.2471/BLT.20.258186

**Published:** 2020-05-01

**Authors:** Bernardo Sousa-Pinto, João Almeida Fonseca, Bruno Oliveira, Ricardo Cruz-Correia, Pedro Pereira Rodrigues, Altamiro Costa-Pereira, Francisco Nuno Rocha-Gonçalves

**Affiliations:** a MEDCIDS – Department of Community Medicine, Information and Health Decision Sciences, Faculty of Medicine, University of Porto, Porto, Portugal.; b CINTESIS – Center for Health Technology and Services Research, University of Porto, Porto, Portugal.

The World Health Organization (WHO) recommends that governments scale up testing for severe acute respiratory syndrome coronavirus 2 (SARS-CoV-2) infection.^1^ Evidence supporting increasing testing comes from the Italian town of Vò,^2^ as well as from countries such as Iceland, Republic of Korea or Norway, where large-scale testing has been identified as one of the reasons behind flattening of the epidemic curve of new infections with a relatively low number of deaths.^3^

Costs of molecular tests, including costs of diagnostic kits, analysis equipment and human resources, may be regarded as an important barrier for scaling up SARS-CoV-2 testing. However, large-scale testing may result in earlier detection of cases, including asymptomatic ones, and thus prevent many new infections and avert hospitalizations. Therefore, financial costs associated with an increased number of tests may in some scenarios be offset by savings in future hospitalization costs. The number of prevented hospitalizations, however, does not simply depend on the number of performed tests. If testing is scaled up, a decrease in the percentage of positive results will ensue, although we cannot predict to which extent, as we are unable to know the number of undetected cases. 

To address this challenge, we developed an online tool^4^ that allows users to simulate different scenarios to project how many hospitalizations (including intensive care unit episodes) could be prevented if more SARS-CoV-2 tests were performed and what would be the underlying economic savings. With this tool, users cannot only simulate different testing frequencies, but also different percentages of positive results. These features allow the projection of additional number of patients that would be expected to be identified, as well as the number of new infections over a defined number of days. This calculation uses the formula *a*(1+*r*)^t^ where *a* corresponds to the additional cases detected by the increased frequency of testing, *r* to the infection growth rate and *t* to the number of days over which the user wishes to project results. Additional required inputs include the number of tested patients, the number of confirmed infection cases, the frequency of hospitalized patients, and the costs of hospitalization and tests. As an example, the tool displays predefined scenarios for different countries in the WHO European Region and the Region of the Americas, along with the corresponding data sources. 

This tool only considers direct costs of testing and hospitalizations, which vary between countries. Prevention of new infections, after testing is scaled up, is expected to prompt additional savings in indirect costs, including decreased productivity losses subsequent to hospital admissions or quarantines. Importantly, less stress on national health systems would allow patients with other diseases to be adequately treated. Since hospitals are being directed to treat patients with COVID-19, some diagnoses and treatments are being cancelled or postponed.^5^^,^^6^ Therefore, further savings and advantages are to be expected if new infections are prevented. Savings may increase in the future, as test production is increased and new technologies become available, reducing the total costs of running tests.

Costs should not be the sole or main reason behind decisions in health care. Nevertheless, data showing that increasing SARS-CoV-2 testing may prompt savings for health-care services, may also persuade decision-makers to scale up testing in a fast and sustained way, and thus meet the recommendation of WHO to increase testing.^1^
